# Comparative transcriptome and metabolome analyses reveal the mechanism of silicon to improve stem rust resistance in oat (*Avena sativa* L.)

**DOI:** 10.1038/s41598-025-21482-9

**Published:** 2025-11-11

**Authors:** Ying-hao Li, Ao Yang, Jun-zhen Mi, Xiao-xia Guo, Lu Tian, Bao-ping Zhao, Jing-hui Liu

**Affiliations:** 1 Science Innovation Team of Oats, Team for Innovation and Utilization of Oat Germplasm Resources, Inner Mongolia Agricultural University, Hohhot, China; 2 Inner Mongolia Academy of Agricultural and Animal Husbandry Science, Hohhot, China

**Keywords:** Oat, Stem rust, Silicon, Physiological, Transcriptome, Metabolome

## Abstract

Silicon (Si) has been demonstrated to enhance oat resistance to stem rust, caused by *Puccinia graminis f. sp. avenae* (*Pga*). However, the molecular mechanisms underlying Si-mediated resistance against *Pga* remain poorly characterized. To address this, we performed transcriptomic and metabolomic analyses on oat plants treated with or without Si and inoculated with *Pga*. Our results showed that Si treatment increased the activities of catalase (CAT), peroxidase (POD), and superoxide dismutase (SOD) under *Pga* infection, thereby inhibiting reactive oxygen species (ROS) accumulation. Transcriptomic analysis identified 143 differentially expressed genes (55 upregulated, 88 downregulated) in Si-treated plants. Most of these genes were associated with diterpenoid biosynthesis, zeatin biosynthesis, and phenylpropanoid biosynthesis. Metabolomic profiling revealed 69 significantly enriched metabolites, including carbohydrates, organic acids, amino acids, and secondary metabolites. Based on KEGG database annotation, these metabolites were primarily involved in arginine biosynthesis; alanine, aspartate, and glutamate metabolism; cyanoamino acid metabolism; aminoacyl-tRNA biosynthesis; pyrimidine metabolism; and purine metabolism. Integrative analysis of transcriptome and metabolome data indicated that Si treatment significantly altered key metabolic pathways in oat leaves, including tryptophan metabolism, glyoxylate and dicarboxylate metabolism, porphyrin metabolism, and chlorophyll metabolism. Collectively, these findings provide novel molecular insights into Si-mediated enhancement of oat resistance to stem rust.

**Supplementary Information:**

The online version contains supplementary material available at 10.1038/s41598-025-21482-9.

## Introduction

Oat (*Avena sativa L*.) is an economically important crop worldwide^1^. It is consumed in different forms such as oatmeal or other whole-grain components, and its high quantities of oil, protein and β-glucan have rekindled attention in recent years^2,3^.

Oat stem rust, caused by *Puccinia graminis f. sp. avenae* (*Pga*), is one of fungal diseases and causes huge yield losses worldwide, especially in the USA and Canada^4^. Many measures, including agronomic measures, host resistance and chemical pesticides, have been taken to reduce the losses caused by stem rust, but these are not always feasible. To date, there is still a lack of reliable and effective methods to control this disease.

Various factors including biotic and abiotic stresses affect the production of oats worldwide and among these factors, stem rust is a significant factor affecting the yield and productivity. Host plant resistance is one of the most effective ways to alleviate the losses caused by *Pga*. Silicon (Si) is the second most abundant element in the Earth’s crust^5^, it has been shown to help plants defend themselves against diseases such as fungus, bacteria, and viruses^6,7^. But the underlying mechanism of action of Si in imparting resistance against fungal diseases has not been understood very well. Three methods have been proposed for Si-enhanced disease and pest resistance. The most typical examples are physical, biochemical and molecular reactions stimulation^6,8^. As a physical barrier, Si deposited under the leaf cuticle or on the surface of cell tissues to mechanically inhibit fungal infection^9^. Increased defense-related enzyme activity, induction of antibacterial compounds, and regulation of systemic signaling agents are some of the biochemical reactions induced by Si in plants^6,8^. Many plants have been shown to contain these mechanisms, such as perennial ryegrass (*Lolium perenne*), wheat (*Triticum aestivum*), and rice (*Oryza sativa*), which protect them from pathogens^10–14^.

The process of Si improving plant disease resistance is quite complex. Many researchers employ cutting-edge molecular technologies to decipher the plant defense mechanisms at the molecular level. On a similar note, for studying the disease resistance mechanism of Si at the molecular level, we proposed that Si plays a role in enhancing the transcription level of the *PR-1* and peroxidase genes, which were found to be responsible for imparting disease resistance in the blast-susceptible rice genotype, *M201*^15^. Si can enhance the host’s resistance to pathogens by inducing plants to produce antibacterial substances, such as antibiotics, lignin, phenols and pathogen specific proteins, which is associated with increased activity of protective enzymes in plants^16^.

Of lately, the combination of transcriptome and metabolome analysis has gained significant popularity for studying the mechanisms of plant defense^17,18^. Previous research have studied the role of exogenous Si application in imparting oat resistance against *Pga* infection at the biochemical and physiological level^19^. In this study, transcriptome and metabolome analyses were combined to identify key genes and metabolites, and ultimately reveal the molecular mechanism of Si mediated resistance towards *Pga* infection in oat.

## Materials and methods

### Experimental material and culture conditions

Oat cultivar Bayou 1 (high susceptible) was used for inoculation with *Pga* (race *TKR*). Twenty oat seeds were grown in a 12 cm diameter pot (12 cm × 15 cm) with the peat soil matrix, seedlings were cultured in a greenhouse (20 ± 2 °C with a photoperiod of 16 h light/8 h dark) at the Oat Research Center of Inner Mongolia Agricultural University.

Two treatments were carried out : (i) Si-P treatment: plants were watered with nutrient solution without soluble Si and inoculated with *Pga* (as a control); (ii) SiP treatment: plants were watered with nutrient solution containing 1.5 mmol L^−1^ potassium silicate and inoculated with *Pga*. In the silicon-deficient treatment, potassium chloride (KCl, pH 5.5) was used to equal the potassium component, and the nutrient solution used was configured according to Hoagland’s classic formula^20^. From the beginning of the emergence of oat seedlings, the corresponding nutrient solutions were used to irrigate the pots every 3 days, 150 mL per pot.

The inoculation was carried out when the oat seedlings grew to the two-leaf stage (one leaf and one sprout). The method of inoculation was carried out as described by Li et al.^21^. First, the leaves were sprayed with a 0.05% Tween-20 solution (Polyoxyethylene sorbitan monolaurate, water soluble emulsifier, 0.05%) using a handheld atomizer to form a water film on the leaves. Then, a flat toothpick (only by contact) was used to pick fresh urediniospores (0.01 g) and inoculated on the seedlings. Finally, the inoculated plants were kept in a mist chamber at 18 to 20 °C for 16 h in darkness. Plants were transferred to a 16/8 h (light/dark) photoperiod, and a climatic chamber at 24 °C with 80 ± 5% humidity.

On the third day after inoculation, fresh leaf tissues were cut and frozen in liquid nitrogen and stored at − 80 °C for physiological analysis, total RNA extraction and metabolomic analysis. Six replicates were used for the metabolomic profiling experiment, whereas three replicates were used for the RNA-seq analysis and physiological analysis. On the eleventh day after inoculation, photographs were taken to record the leaf phenotype of the plants.

### Physiologycal parameters

The activities of superoxide dismutase (SOD), peroxide (POD), catalase (CAT) and phenylalanine ammonialyase (PAL) were measured using Assay Kit YX-C-A500, YX-C-A502, YX-C-A501, and YX-C-A604 respectively (Sino Best Biological Technology Co., Ltd., China). The contents of H_2_O_2_ and O_2_^−·^ were measured using Assay Kit YX-C-A407 and YX-C-A400 respectively (Sino Best Biological Technology Co., Ltd., China). We performed a One-Way ANOVA analysis using SPSS software (IBM SPSS Statistics, Version 19.0, IBM, Beijing, China) to assess the associations between different indices and the treatments.* P* ≤ 0.05 was considered significant.

### Metabolite extractions

Samples (80 mg) were accurately transferred to a 1.5 mL Eppendorf tube. Two small steel balls were added to the tube. 20 μL of L-2-chlorophenylalanine (0.3 mg·mL^−1^) dissolved in methanol was used as the internal standard. To each sample, 1 mL of a methanol and water mixture (7:3, v/v) was added, and the mixture was placed at − 20 °C for 2 min, followed by 2 min of grinding at 60 Hz. The whole samples were ultrasonically extracted in an ice-water bath for 30 min. After centrifugation at 4°C (13,000 rpm) for 10 min, 150 μL of the supernatant was taken with a crystal syringe, filtered through a 0.22 μm microfilter, and loaded into LC vials. Vials were stored at − 80 °C until LC–MS analysis. QC prepared a mixed sample by combining aliquots from all samples.

### Data preprocessing and statistical analysis

Baseline filtering, peak identification, integration, retention time correction, peak alignment and normalization of raw LC–MS data were performed using Progenesis QI V2.3 software (Nonlinear, Kinetics, Dynamics, Newcastle, UK).

In order to observe the overall distribution among samples and the stability of the whole analysis process, a matrix was introduced into R and principal component analysis (PCA) was performed. Orthogonal partial least squares discriminant analysis (OPLS-DA) and partial least squares discriminant analysis (PLS-DA) were used to identify the differential accumulated metabolites (DAMs) among the groups. To prevent overfitting, sevenfold cross-validation and 200-response permutation testing (RPT) were employed to assess the model’s quality.

Variable importance of projection (VIP) values were used to rank the overall contribution of each variable to the OPLS-DA model. A two-tailed Student’s t-test was used to determine whether there were significant differences in metabolites between the groups. DAMs were screened with VIP ≥ 1.0 and *P*-values ≤ 0.05. The enrichment analysis of DAMs using the KEGG database was conducted, with a significance threshold set at *P* ≤ 0.05.

### RNA extraction, cDNA library construction, and transcriptome sequencing

Total RNA was extracted from the oat leaves infected by Pga using mirVana™ miRNA ISOlation Kit. Sequencing libraries were created using the TruSeq Stranded mRNA LT Sample Prep Kit following the manufacturer’s instructions. Samples (1 μL) were used to check the size and purity of the library with Agilent 2100 Bioanalyzer. Trimmomatic was used to process raw data^22^. In order to obtain the clean reads, the reads containing ploy-N and the low-quality reads were deleted. Hisat2 was used to map clean reads to the reference genome^23^.

Reference gene source: Avena sativa (oats); reference genome source: https://wheat.pw.usda.gov/GG3/graingenes_downloads/oat-ot3098-pepsico (accessed on 1 July 2021).

### Gene annotation, differential expression, and enrichment analyses

Gene functions were annotated based on the following databases: COG (http://www.ncbi. nlm.nih.gov/COG/, accessed on 1 March 2022); Nr (http://www.ncbi.nlm.nih.gov/, accessed on 1 March 2022); GO (http://www.geneontology.org/, accessed on 1 March 2022); Swiss-Prot (http://www.uniprot.org/, accessed on 1 March 2022); KOG (http://www.ncbi.nlm.nih.gov/KOG/, accessed on 1 March 2022) and KEGG (http://www.genome.jp/kegg/, accessed on 1 March 2022)^24–26^.

The FPKM value of each gene was calculated by cufflinks, and the read count of each gene was calculated by htseq-count. The DESeq R package functions were used to identify differentially expressed genes (DEGs) and estimate the size factor and nbinom test. *P*-value ≤ 0.05 and |log_2_FC|≥ 1 was the threshold value of significantly differential expression. Cluster analysis of DEGs was used to study genes expression pattern. Based on the hypergeometric distribution, GO and KEGG pathway enrichment analysis were performed using R (version 3.6.2).

### Pearson correlation analysis and co-expression analysis

The pearson correlations between DEGs and DAMs were calculated using cor function of R. DEGs and DAMs were simultaneously mapped to the KEGG pathway database to identify common pathways using a tool developed by OE Biotech Co., Ltd. Cytoscape software was used to establish a co-expression network consisting of DEGs and DAMs.

### Real-time quantitative reverse transcription PCR analysis

Eight genes were analyzed in this study, and actin was use as the reference gene. Total RNA was extracted from the oat leaves infected by *Pga* using Ambion-1912 according to the manufacturer’s instructions. Reactions were carried out at 94 °C for 30 s in 384-well optical plates (Roche, Switzerland), followed by 45 cycles at 94 °C for 5 s and 60 °C for 30 s. For each sample, three reactions were performed. Primers were designed and synthesized by TsingKe Biotech (Table S1). The mRNAs expression levels were normalized to the actin expression and calculated using the 2^−ΔΔCt^ method.

## Results

### Phenotypes

**Fig. 1:**
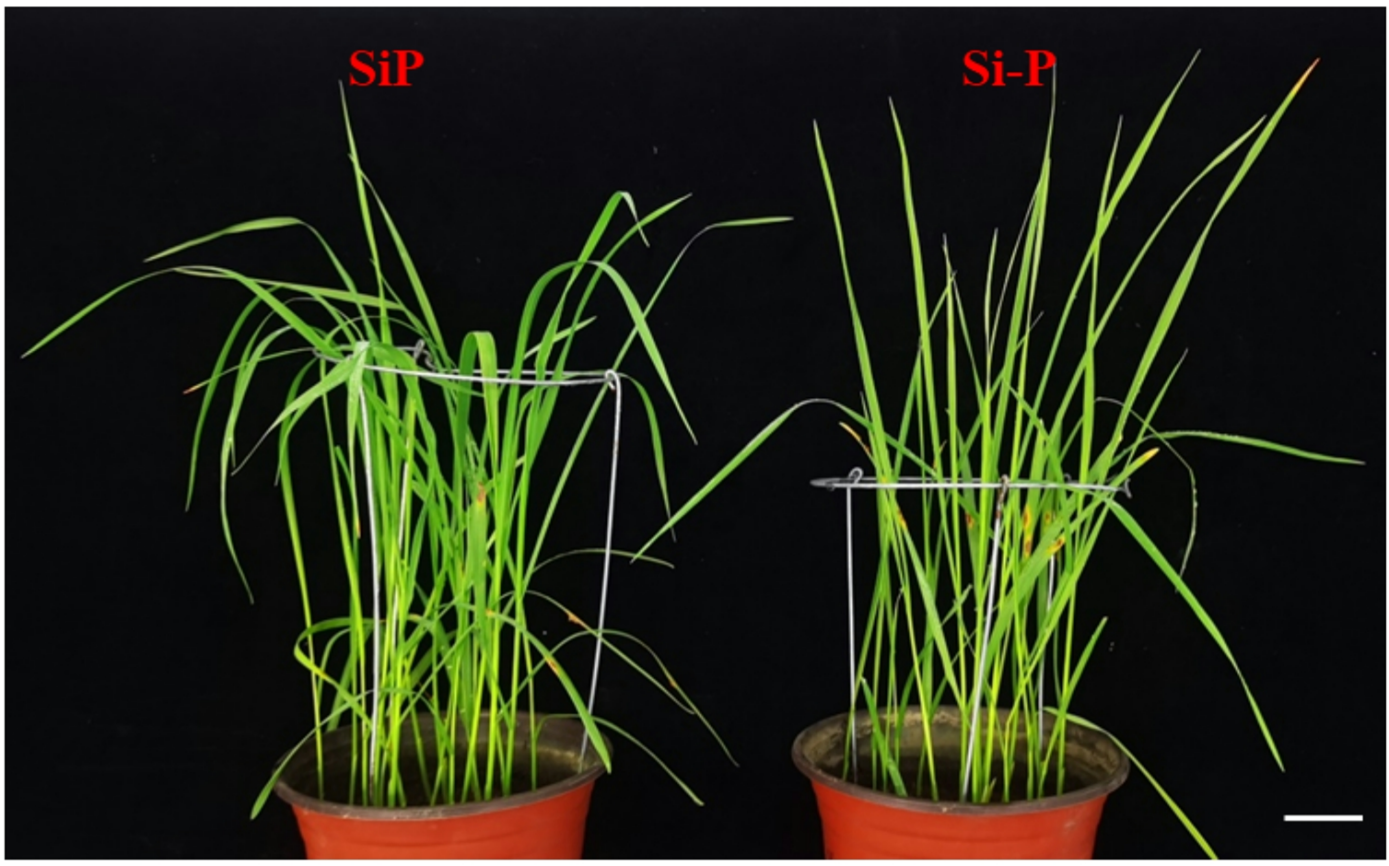
Effects of Si application and *Pga* inoculation on phenotype of oat, Scale bar = 1 cm. Si-P, *Pga* inoculation; SiP, *Pga* inoculation and 1.5 mmol L^–1^ Si application.

Clearly, 11 days after inoculation with *Pga*, rust symptoms appeared as bands of pustules on oat leaves without Si, which were significantly reduced after Si treatment (Fig. 1). It is important to note that the effect of Si treatment on *Pga* resistance (the highest disease level and severity) has been previously published and is now referenced in this article (Table 1).

**Table 1:** Effects of silicon on control efficacy of oat to stem rust^27^.

Treatment	Highest disease level	Severity (%)
Si-P	4 (Highly infection)	7.02 ± 0.21 a
SiP	2 (Moderate resistance)	4.52 ± 0.16 b

### Physiological changes

**Fig. 2:**
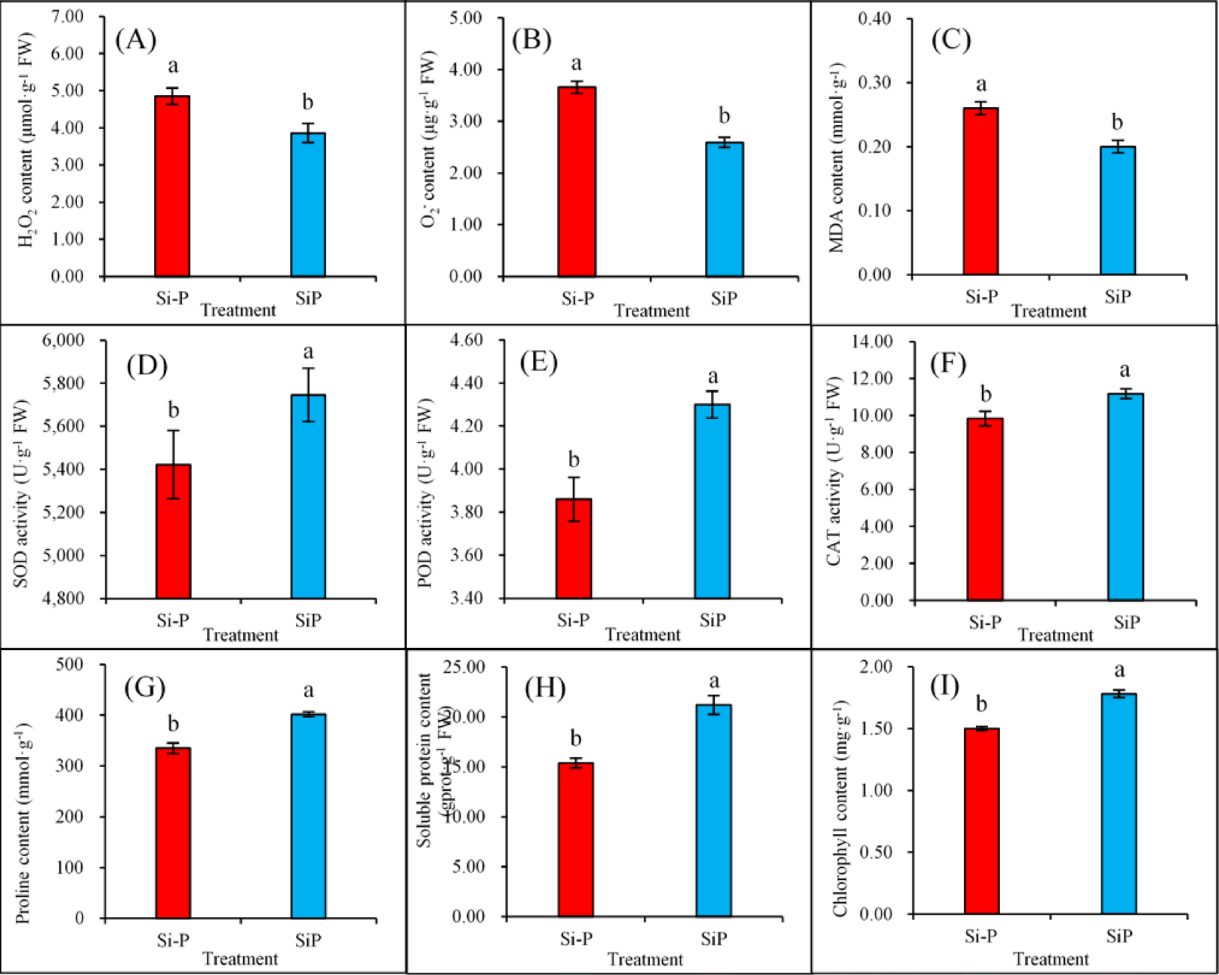
Effects of Si application and *Pga* inoculation on physiological changes of oat leaves. H_2_O_2_ content (**A**),O_2_^−^ content (**B**), CAT activity (**C**), SOD activity (**D**), POD activity (**E**), MDA content (**F**), Proline content (**G**), Soluble protein content (**H**), Chlorophyll content (**I**). Data are expressed as mean ± SE (n = 6). The error bars represent the standard deviations. Different letters above the bars indicate significant differences (*P* ≤ 0.05). Si-P, *Pga* inoculation; SiP, *Pga* inoculation and 1.5 mmol L^−1^ Si application.

When plants experience disease stress such as *Pga* infection, silicon (Si) can induce cellular physiological responses and activate systemic plant defenses. Under *Pga* infection, Si treatment significantly reduced the contents of H_2_O_2_ (Fig. 2A), O_2_^−^ (Fig. 2B), and MDA (Fig. 2C) by 20.41%, 29.23%, and 23.08%, respectively (*P* ≤ 0.05). Conversely, it increased the activities of SOD (Fig. 2D), POD (Fig. 2E), and CAT (Fig. 2F) by 5.98%, 11.40%, and 13.62%, respectively (*P* ≤ 0.05). Furthermore, Si treatment also significantly increased the contents of proline (Fig. 2G), soluble protein (Fig. 2H), and chlorophyll (Fig. 2I) by 19.94%, 37.84%, and 18.67%, respectively (*P* ≤ 0.05).

### Metabolite profile

The differences in metabolome between Si-P and SiP treatment were studied using a combination of LC/MS detection platform, a self-made database, and multivariate statistical analysis. A total of 21,482 metabolites were found between Si-P and SiP treatment (Table S2), which could be roughly divided into several major classes, such as amino acids and their derivatives, predominantly organic acids, nucleotides, indoles, phenylpropanoids, and lipids.

**Fig. 3:**
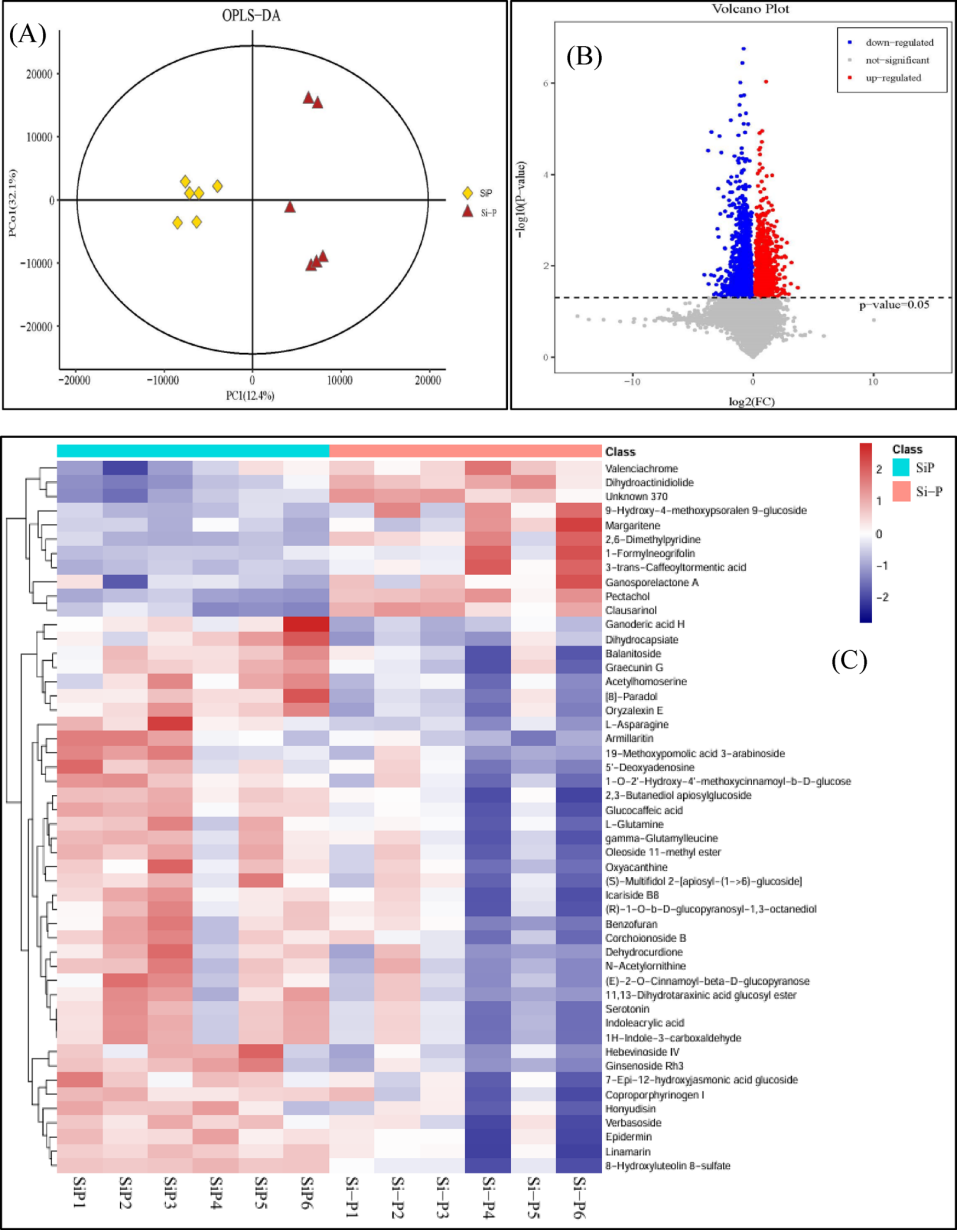
(**A**) Principal component analysis of expressed metabolites. (**B**) Volcano Plot of DAMs between Si-P and SiP. The blue dots represent downregulated DAMs, the red dots represent upregulated DAMs, and the gray dots represent the insignificant DAMs. (**C**) Heatmap hierarchical clustering of DAMs. Red indicates upregulated DAMs, and blue indicates downregulated DAMs. Si-P, *Pga* inoculation; SiP, *Pga* inoculation and 1.5 mmol L^–1^ Si application.

A series of paired OPLS-DA models was used to maximize the distinction between samples and to focus on metabolic changes that contribute significantly to the classifications of the results. The OPLS-DA differences between Si-P and SiP treatment demonstrated that these samples had significant biochemical perturbation, indicating that the results are reliable (Fig. 3A). So, subsequent analysis was performed.

Table S3 and Fig. 3B list all DAMs (*P* ≤ 0.05) with variable significance in the projection (VIP ≥ 1.0), there were 69 DAMs (55 upregulated and 14 downregulated) between Si-P and SiP treatment. We generated a heatmap of the 50 DAMs with the highest VIP values to characterize the changes of DAMs (Fig. 3C).

**Fig. 4:**
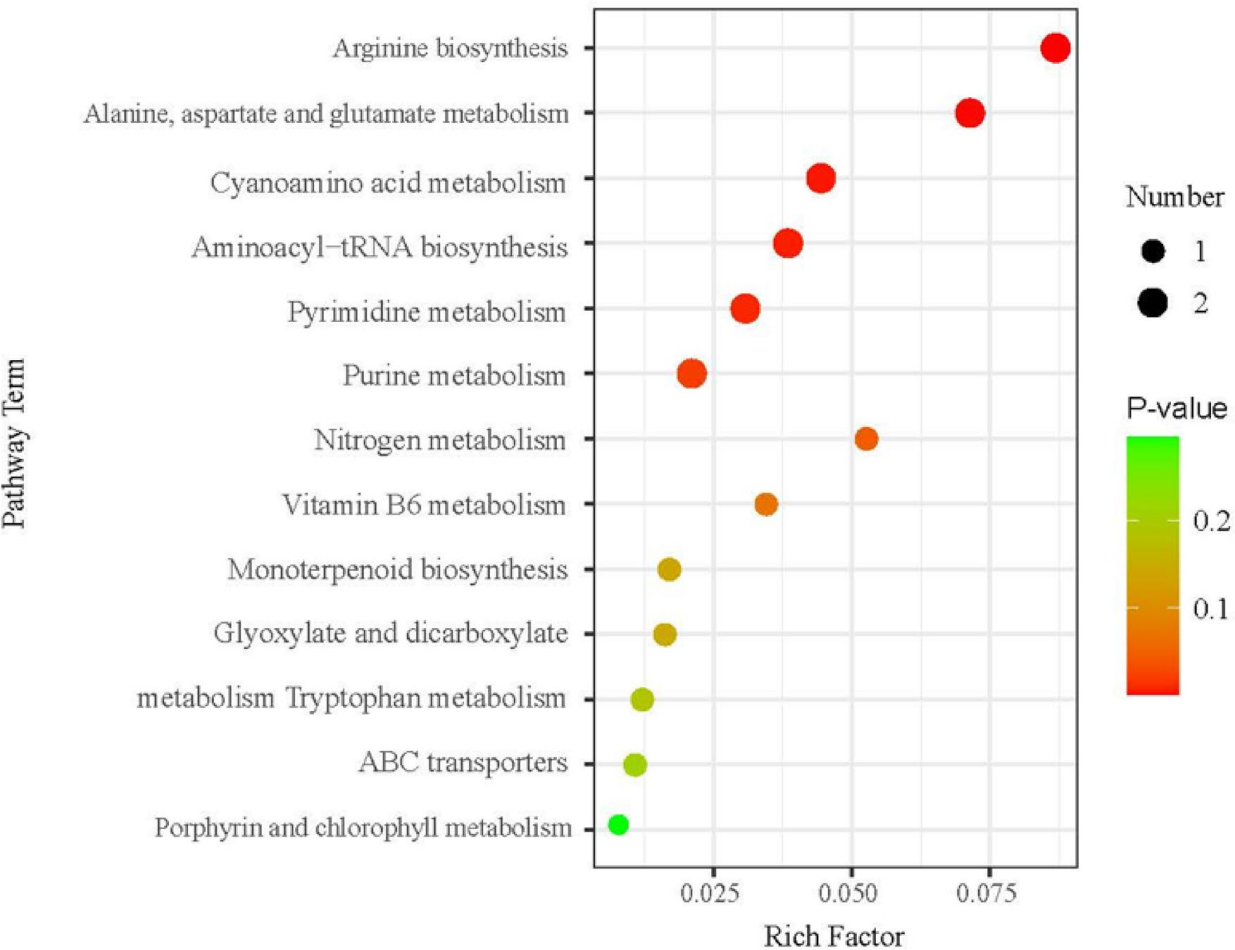
KEGG pathway enrichment analysis of DAMs between Si-P and SiP. The larger the enrichment factor, the greater the enrichment degree. The size of the dot in the Fig. indicates the number of DAMs enriched in the pathway, and the color of the dot indicates the size of the *P*-value. Higher *P*-values correspond to lower enrichment levels. Si-P, *Pga* inoculation; SiP, *Pga* inoculation and 1.5 mmol L^–1^ Si application.

The KEGG database was used to functionally annotate DAMs (Table S4). The most enriched pathways were observed to include arginine biosynthesis, alanine, aspartate and glutamate metabolism, cyanoamino acid metabolism, amino-acyl-tRNA biosynthesis, pyrimidine metabolism and purine metabolism (Fig. 4).

### RNA sequencing and identification of differentially expressed genes

**Fig. 5:**
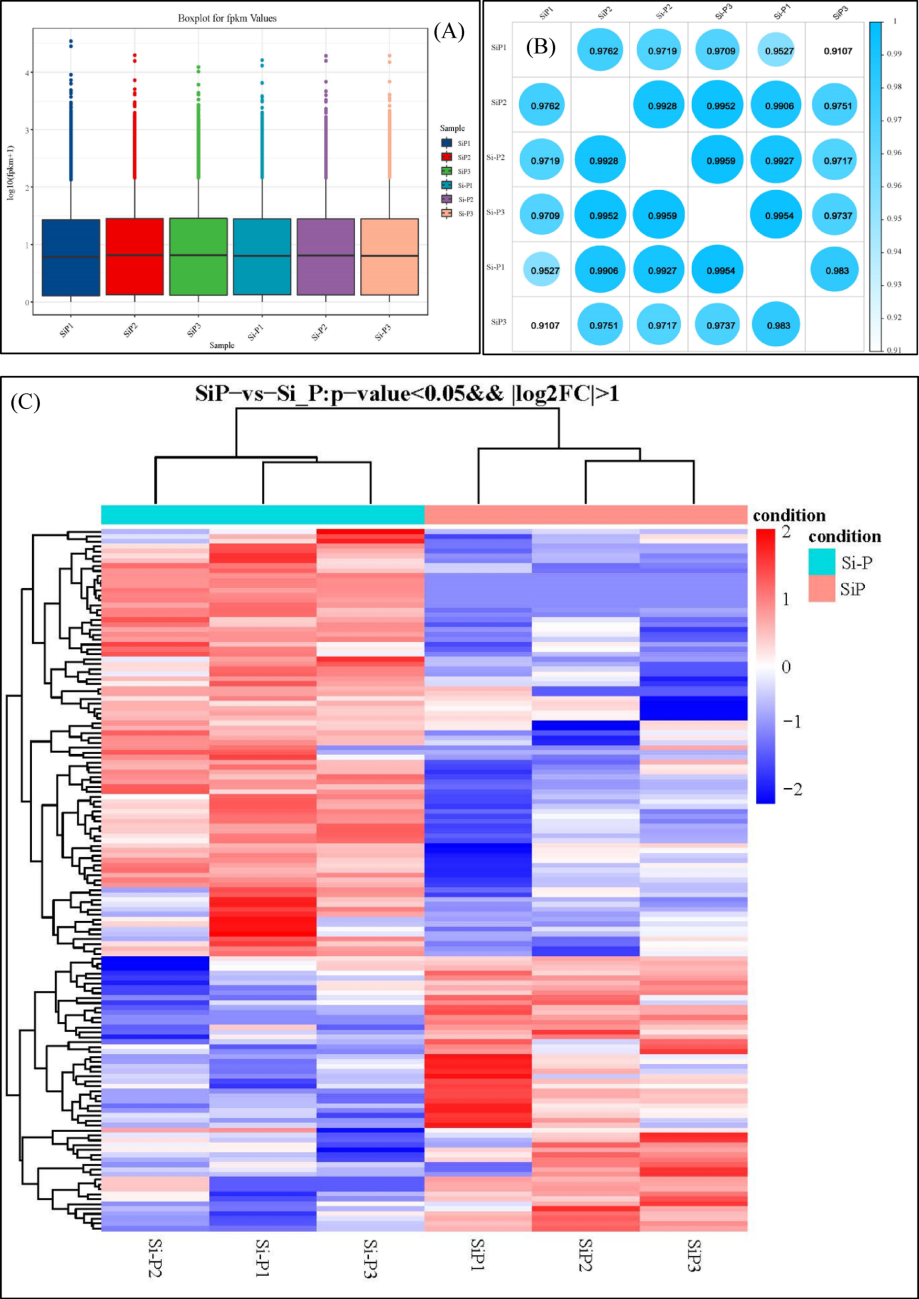
(**A**) The boxplot of FPKM under different experiment conditions. The X-axis is the sample name and the Y-axis is log_10_ (FPKM) value. The boxplot for each region corresponds to five statistics (top to bottom are maximum, third quartile, median, first quartile and minimum). (**B**) The heatmap of correlation coefficient between samples. (**C**) The heatmap of DEGs in oat leaves between Si-P and SiP. Red indicates upregulated DEGs, blue indicates downregulated DEGs. Si-P, *Pga* inoculation; SiP, *Pga* inoculation and 1.5 mmol L^–1^ Si application.

**Fig. 6:**
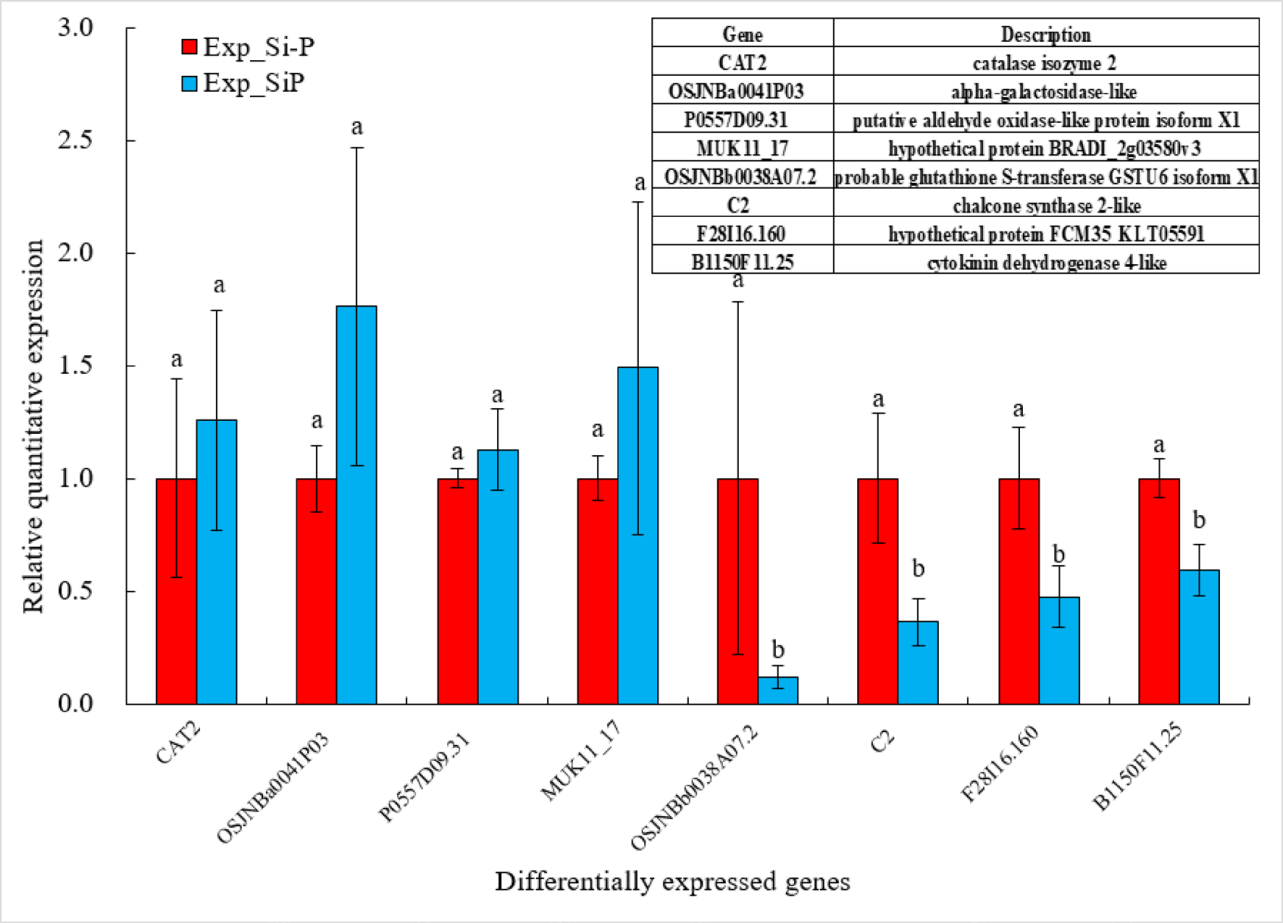
Gene expression analysis of DEGs by qRT-PCR. Si-P, *Pga* inoculation; SiP, *Pga* inoculation and 1.5 mmol L^–1^ Si application. Data given in the form of mean ± SE.

The contigs were employed in transcriptome assembly, and a summary of the FPKM data is provided in Fig. 5A. For each treatment, the biological replicates had a high correlation (R^2^ > 0.95) (Fig. 5B). On the basis of this information, we could infer that the biological replicates were highly reliable. Heat map analysis showed all significantly DEGs (*P* ≤ 0.05 and Fold Change ≥ 2) between Si-P and SiP treatment are listed in Table S5 and Fig. 5C. In total, 143 genes were differentially expressed, with 55 upregulated and 88 downregulated, suggesting that treatment with exogenous Si may improve the *Pga* resistance in oat through altering the expression of these genes. Finally, ten genes between Si-P and SiP treatment were randomly selected for quantitative RT-PCR. The results showed that qRT-PCR data were in good agreement with RNA-Seq profiles (Fig. 6).

**Fig. 7:**
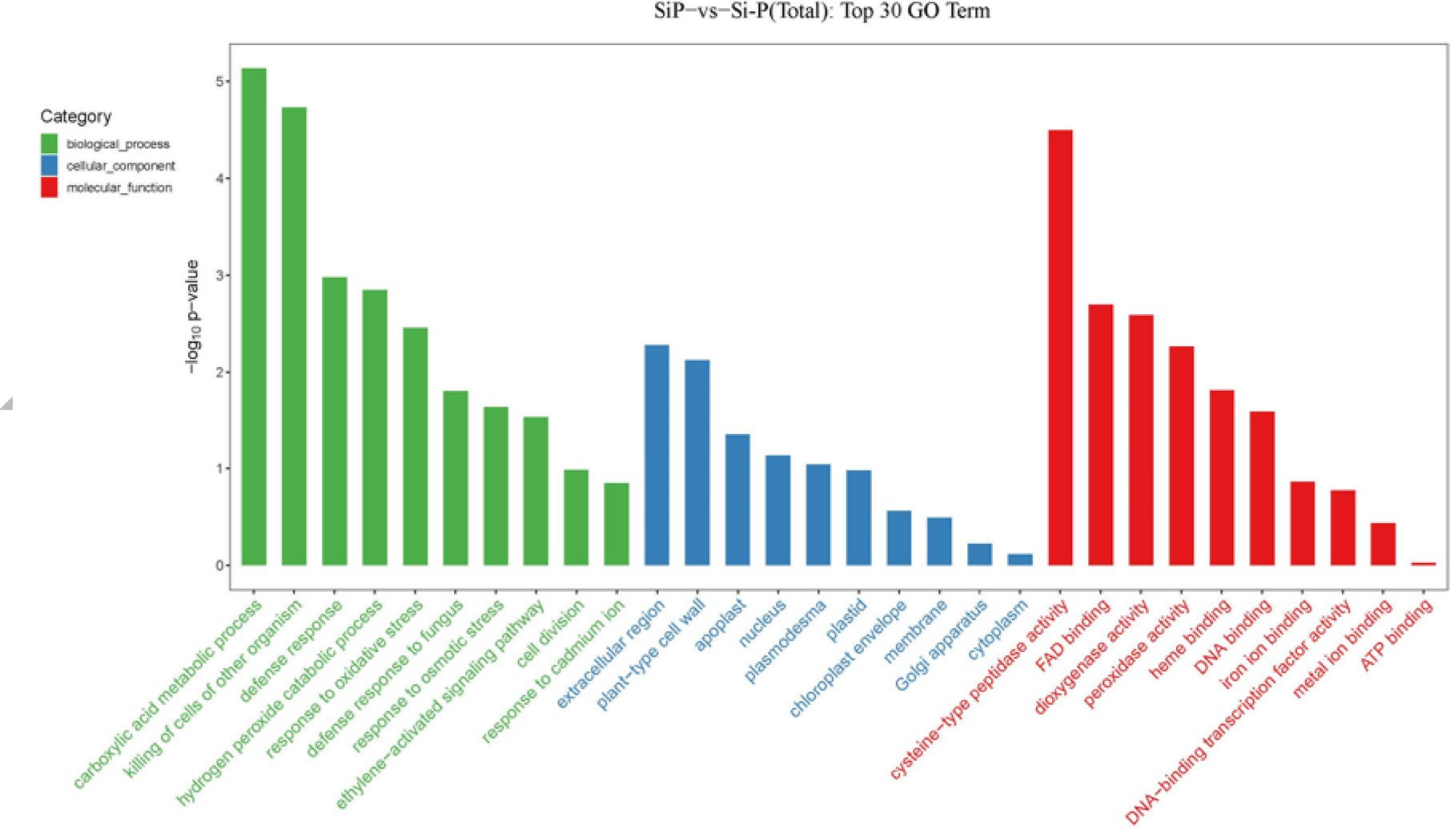
GO analysis of differentially expressed genes (DEGs) between Si-P and SiP. Si-P, *Pga* inoculation; SiP, *Pga* inoculation and 1.5 mmol L^–1^ Si application.

**Fig. 8:**
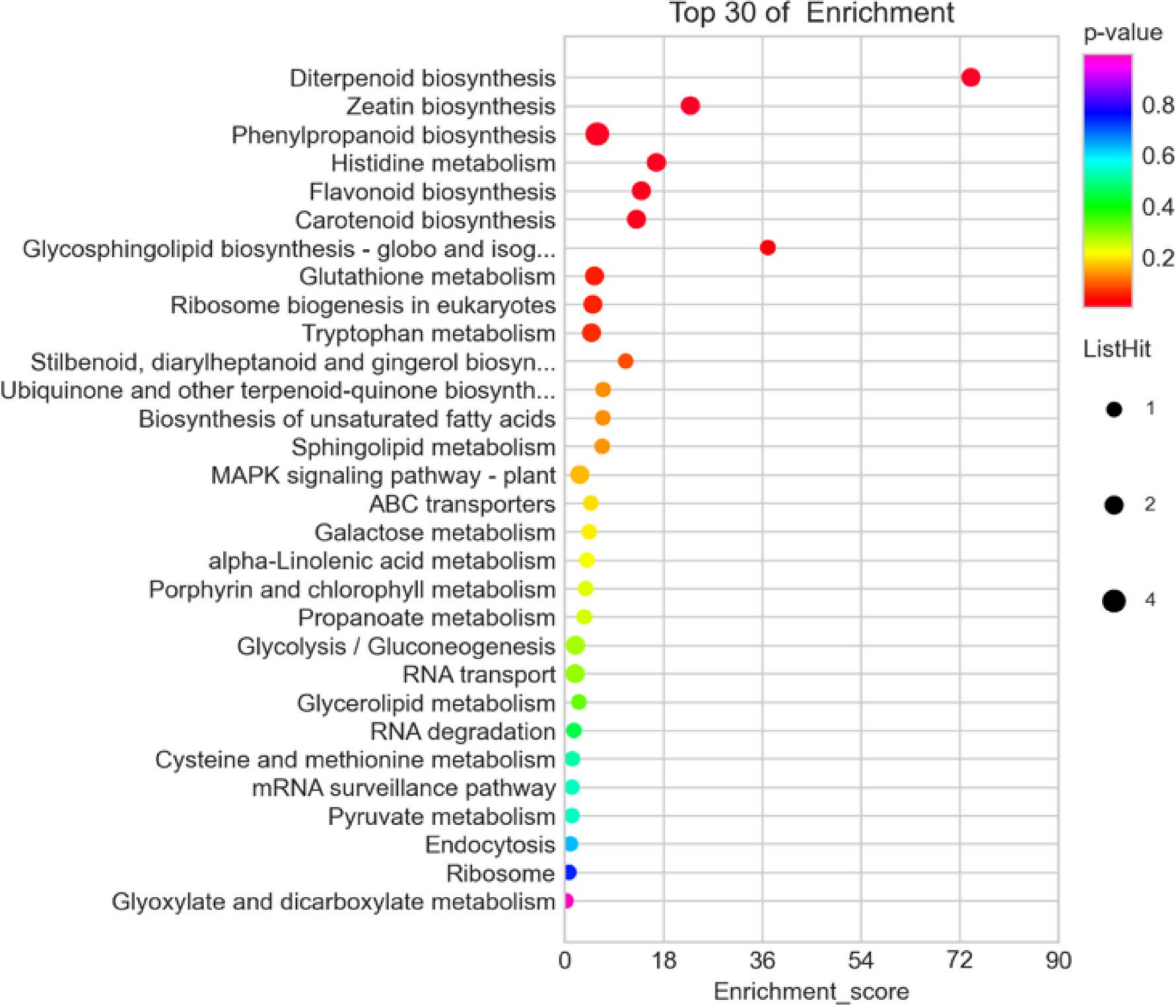
KEGG pathway enrichment analysis of DEGs between Si-P and SiP. The X-axis represents the enrichment score. The size of the bubble indicates the number of DEGs enriched in the pathway, and the color of the bubble indicates the magnitude of the *P*-value. The smaller the *P*-value, the greater the significance. Si-P, *Pga* inoculation; SiP, *Pga* inoculation and 1.5 mmol L^–1^ Si application.

To classify the functions of the DEGs between Si-P and SiP treatment, a GO analysis was performed. The DEGs were significantly enriched in terms of carboxylic acid metabolic process, killing of other organisms’ cells, and defense response within the biological process categories. The extracellular region, plant-type cell wall, and apoplast were significantly enriched within the cellular component category. The DEGs reveal significant enrichments in categories for cysteine-type peptidase activity within the molecular function category, and most of the differentially expressed DEGs were enriched in the cellular component category (Fig. 7, Table S6).The KEGG enrichment results were analyzed as a scatter plot using KEGG function annotations (Table S7). Diterpenoid biosynthesis, zeatin biosynthesis and phenylpropanoid biosynthesis were the top three significantly enriched KEGG pathways between Si-P and SiP treatment (Fig. 8).

### Integrated analysis of the transcriptome and metabolome

**Fig. 9:**
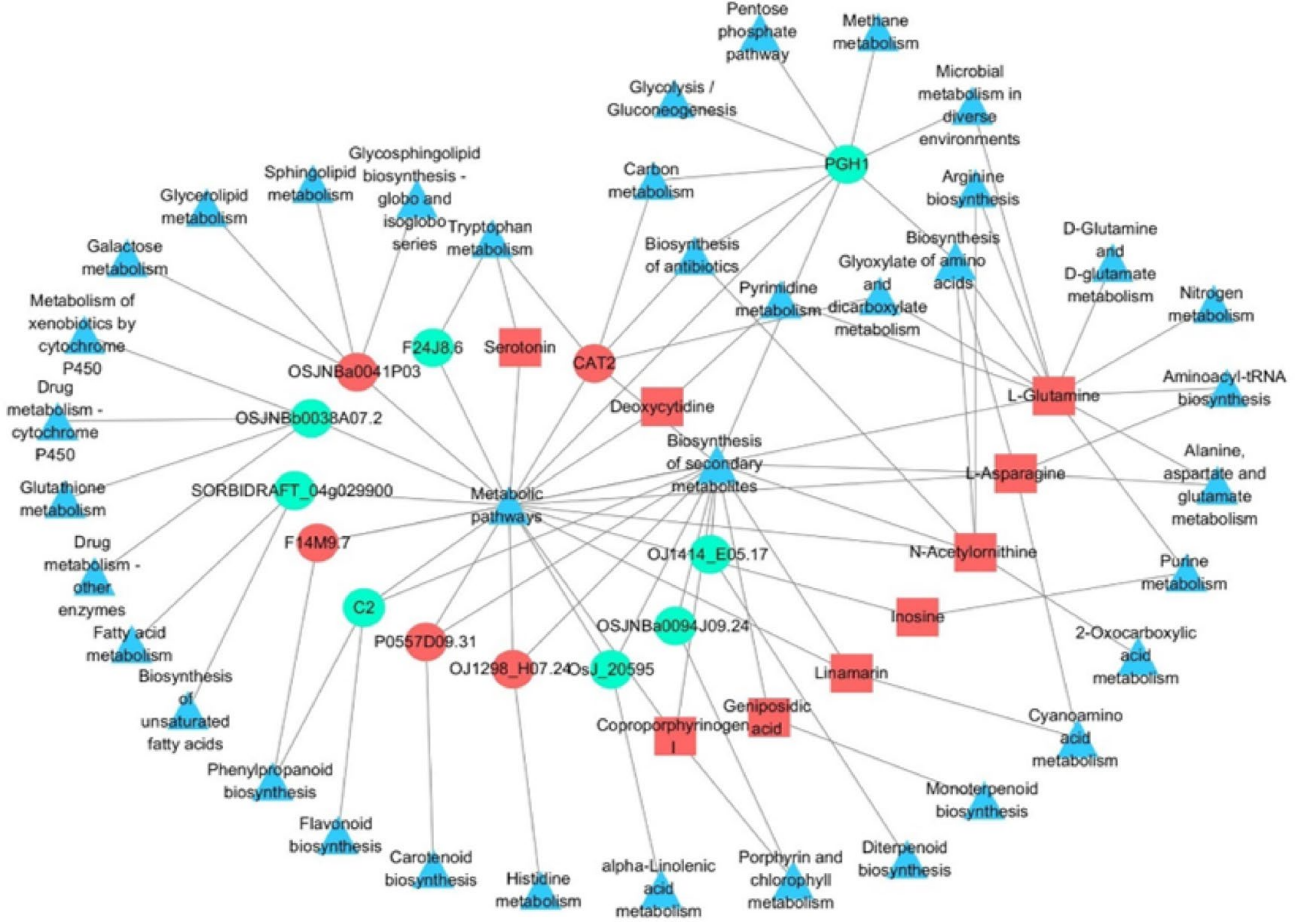
Co-expression network of DEGs and DAMs.

We identified the gene regulatory network associated with Si application under Pga infection using an integrated analysis of the transcriptome and metabolome. Except for the Zeatin biosynthesis pathway, a total of 38 pathways were affected by both DEGs and DAMs (Table S8), and these pathways could be linked to each other through differential factors (Fig. 9). In addition to metabolic pathways, biosynthesis of secondary metabolites contained the most differential factors, most of which were significantly downregulated after Si application under *Pga* infection conditions, and this pathway interacted with other pathways simultaneously, such as cysteine and methionine metabolism, purine metabolism, phenylpropanoid biosynthesis, porphyrin and chlorophyll metabolism.

Triangle, squares and circles represent pathways, DAMs and DEGs, respectively. For DEGs and DAMs, red and green indicate the upregulated and downregulated factors, respectively.

## Discussion

Silicon has been shown to be effective in controlling plant diseases caused by fungus, such as rust^28^. In our previous study, it has been confirmed that Si (1.5 mmol L^−1^) can enhance the resistance of oat stem rust by improving the photosynthetic performance and antioxidant capacity of leaves^19^. However, Si-mediated pathogen resistance is a complicated phenomenon that requires further molecular investigation. An integrated analysis of the transcriptome and metabolome revealed significant differences in gene expression and metabolite levels between Si-P and SiP treatments, which may provide insight into the Si-mediated oat defensive response. Transcriptomic, metabolomic, and proteomic analyses have recently been used to explain the plant-pathogen interactions in a variety of plants^29–31^.

After Si treatment, the increase of plant resistance to pathogens is due to the accumulation and polymerization of silicic acid in the host cell wall, which makes it more resistant to pathogen penetration^32^. We discovered that the extracellular area, plant-type cell wall, and apoplast were considerably enriched within the cellular component category using GO analysis to define the activities of the DEGs. Kauss et al.^33^ showed that in the process of inducing systemic acquired resistance (SAR) in cucumber, Si treatment resulted in upregulation of genes encoding proline proteins, which could prevent fungi from penetrating the cell wall of epidermal cells. In this study, Si application induced the expression of genes encoding proline synthesis, which prevented *Pga* from invading the epidermal cell wall of oat leaves. On one hand, proline cross-links with lignin to enhance the mechanical strength of plant cell walls, promoting rapid deposition at fungal invasion sites and physically blocking hyphal penetration.

On the other hand, proline activates the phenylpropanoid metabolism pathway, stimulating the synthesis of antifungal compounds such as lignin and flavonoids.

**Fig. 10:**
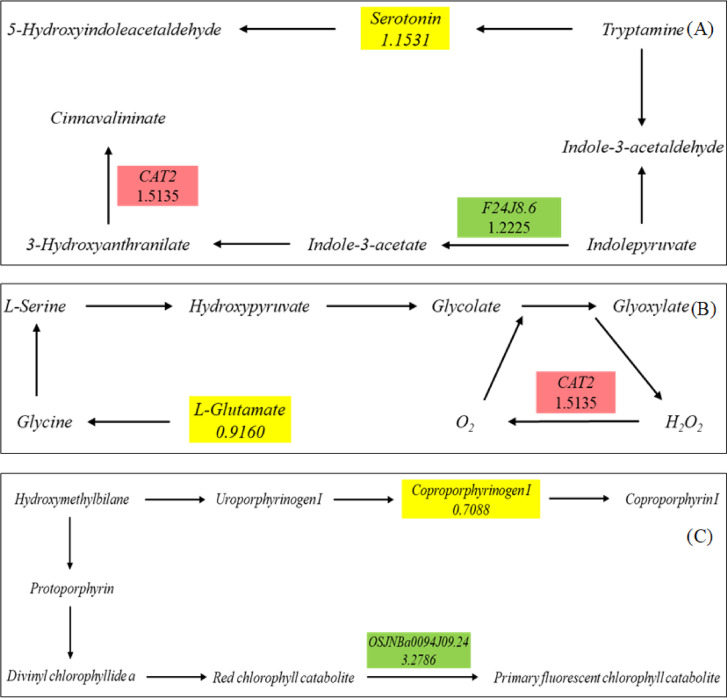
Networks of the ‘Tryptophan metabolism’ (**A**), ‘Glyoxylate and dicarboxylate metabolism’ (**B**), and ‘Porphyrin metabolism’ (**C**).

Plant defense responses against pathogens need a steady supply of energy, mostly from fundamental metabolic activities^34^. Following pathogen detection and signal transduction processes, primary metabolites, such as amino acids, also operate as signaling molecules to induce defense responses^35,36^. Amino acids contribute to the inhibition of pathogen infection and participate in the regulation of intracellular signal transduction pathways, implying that amino acid plays a key role element in plant resistance to pathogens^37,38^. Our results discovered that Si treatment promoted the significant accumulation of amino acids such as L-glutamine, N-acetylornithine, and L-asparagine. Tryptophan is an essential amino acid in mammals that produces proteins, neurohormones, serotonin and the vitamin niacin. In addition, it is also a precursor for auxin and a variety of secondary metabolites that protect plants from fungal and bacterial attack^39,40^. Our transcriptome and metabolome data revealed that Si treatment activated the tryptophan metabolism pathway within oat leaves against *Pga* infection, and serotonin was significantly upregulated (Fig. 10A). Researchers have discovered that serotonin generated from tryptophan plays a vital function in rice’s resistance to insects^41^. As a by-product of tryptophan metabolism, serotonin is involved in systemically acquired resistance and programmed cell death in Arabidopsis, and can promote plant growth and defensive response^42,43^. In our study, transcriptome analysis showed that catalase-related gene (CAT2) was upregulated after Si treatment. Notably, Si treatment could also reduce H_2_O_2_ accumulation and improve leaf catalase activity under *Pga* infection conditions. Similarly, Si significantly increased the activity of catalase and other pathogenesis-related proteins (PRPs), and effectively inhibited the expansion of asparagus stem blight^44^. Under the normal aerobic conditions, the metabolic byproduct of *Pga* may cause the creation of reactive oxygen species (ROS), which are more reactive than molecular oxygen and can harm cellular components^45^. It is concluded that Si was involved in *Pga* resistance by regulating serotonin in the tryptophan metabolism pathway.

Glutamine is essential for ammonia detoxification, acid–base balance, nitrogen metabolism, cell signaling, osmotic control and other processes in plants^46,47^. Because glutamine serves as a key substrate for a variety of metabolic pathways, it is an essential amino acid for the regular functioning of living organisms. It also plays a role in the growth and pathogenicity of plant fungal diseases^46^. In our research, combined transcriptome and metabolome analysis indicated that Si treatment activated the glyoxylate and dicarboxylate metabolism pathway under *Pga* infection conditions, and glutamine was significantly upregulated (Fig. 10B). Si treatment increased HvST1;1 transcript level in barley roots, which increased NO_3_^−^ uptake. Higher concentration of NO_3_^−^ increased the level of glutamine in tandem, allowing the plant to cope against osmotic stress and S shortage^48^.

After pathogen infection, the total amount of chlorophyll in leaves decreased, which affected the transmission, absorption and conversion of light energy between photosystem I and photosystem II, and ultimately reduced the photosynthetic rate. D'Addazio et al.^49^ found that Si improved the chlorophyll content of black pepper plants infected by *Fusarium solani f. sp. piperis.* Our study indicated that Si significantly increased the chlorophyll content of oat leaves. The result of this study showed that a gene (OSJNBA0094J09.24) encoding red chlorophyll catabolite reductase was downregulated, indicating that Si treatment promoted the accumulation of chlorophyll in porphyrin and chlorophyll metabolism pathways (Fig. 10C). Gou et al.^50^ reported that Si application increased the concentration of uroporphyrinogen and the levels of chlorophyll and its precursor (protoporphyrin) under nitrate stress. In this study, Si treatment increased the level of coproporphyrinogen (Fig. 10C). These results suggest that Si could improve oat resistance to stem rust by enhancing chlorophyll synthesis.

Si treatment enhances oat resistance to *Pga* by modulating antioxidant enzyme activities, upregulating genes involved in zeatin biosynthesis, diterpenoid biosynthesis, and phenylpropanoid biosynthesis, and promoting metabolite accumulation (including carbohydrates, amino acids, and organic acids). Additionally, Si activates tryptophan, glyoxylate and dicarboxylate, porphyrin, and chlorophyll metabolism. These findings advance our molecular-level understanding of Si-mediated resistance to stem rust in oats.

The substances on the line are genes, and the locations of the nodes indicate metabolites. Red and green indicate upregulated and downregulated genes, respectively; yellow indicates upregulated metabolites.

## Conclusions

In summary, Si treatment may enhance oat resistance to *Pga* by modifying antioxidant enzyme activities, upregulating the expression of many genes involved in diterpenoid biosynthesis, zeatin biosynthesis and phenylpropanoid biosynthesis, promoting the accumulation of metabolites, including carbohydrates, amino acids and organic acids, as well as the activation of tryptophan, glyoxylate and dicarboxylate, porphyrin and chlorophyll metabolism. Our findings contributed to a better understanding of Si-mediated oat stem rust resistance at the molecular level.

## Supplementary Information

Supplementary Information.

## Data Availability

All the original sequencing data in this study were stored in the NCBI database with the login number “PRJNA810908” (https://dataview.ncbi.nlm.nih.gov/object/PRJNA810908, accessed on 1 October 2021). The datasets used and/or analysed during the current study available from the Science Data Bank database (https://www.scidb.cn/en/s/nmeyUr).
